# Going beyond the pandemic: ‘next generation eu’ and the politics of sub-regional coalitions

**DOI:** 10.1057/s41295-022-00302-8

**Published:** 2022-05-04

**Authors:** Sergio Fabbrini

**Affiliations:** Department of Political Science, Luiss University, Viale Romania, 32 - 00197 Rome, Italy

**Keywords:** Pandemic, Fault lines, Sub-regional coalitions, Next Generation EU, Discursive institutionalism, Segmentation

## Abstract

The article aims to explain the 2020 approval of ‘Next Generation EU’, the program for helping the EU member states to go beyond the pandemic. The approval of NG-EU is interpreted in the context of a confrontation between three distinct interstate coalitions, coordinating a group of countries from the north (the Frugal coalition) against the core of continental countries (the Solidarity coalition) and then a group of countries from the east (the Sovereignty coalition) against the previous two coalitions allied together. Based on the discursive institutionalism’s approach, the article reconstructs the policy discourse shared by the members of each coalition, coherently utilized along the fault lines which conceptually structured the 2020 policy-making process. The policy coherence and the organizational consistency of the three coalition cores affected the EU policy-making process more than the inter-institutional relations between the Commission and national governments. The article concludes advancing arguments for interpreting the sub-regional segmentation of the EU.

## Introduction

‘Next Generation EU’ (NG-EU) is the crucial programme, decided by the EU, for helping its member states to go beyond the pandemic. How to explain it? Here, I consider the deliberation (policy claims) and decision-making (policy outcome) phases of the policy-making process which started on March 2020 and concluded on 16 December 2020 with the adoption by the Council of ministers (hereinafter only Council), with the European Parliament (EP)’s consent, of the regulation providing for the Multiannual Financial Framework (MFF) 2021–2027 of €1,074.3 billion for the EU27, to which was connected the NG-EU recovery instrument of €750 billion decided by the Council decision 2020/2053. There is a wide consensus that NG-EU “constituted an unprecedented integrative step for the EU since it involved the European Commission undertaking massive borrowing on the capital market for the first time” (Ferrera et al. 2021: p. 13).

After the initial difficulty in acknowledging the pandemic’s magnitude, from March to December 2020 (the period after December 2020 is not relevant for my research question), an unprecedented political battle developed among the leaders of the EU member states and its institutions. A battle which lined up a group of countries from the north (The Netherlands, Austria, Denmark, Sweden and later Finland, the Frugal coalition) against a large group of countries at the core of the EU (France, Italy, Spain, Portugal, Greece, Ireland, Belgium, Luxembourg, and Slovenia, later joined by Germany, the Solidarity coalition) and, finally, two countries from the east (Poland and Hungary, the Sovereignty coalition) against the rest of the EU. The coalitions are named according to their own self-definition (in the case of the northern group) or for the recurrence of the values each group mobilized during the policy-making process (solidarity or sovereignty, in the cases of the other two coalitions), and not according to a normative assessment. The division between the Frugal and the Solidarity coalitions emerged regarding three fault lines (the interpretation of the crisis, the resources needed to go beyond the latter and the governance for dealing with the post-pandemic recovery). The outcome of this division was the NG-EU programme, connected to the MFF 2021–2027, and the rule of law conditionality regime agreed by the Council and the EP for the use of the corresponding funds. The rule of law conditionality brought to the surface a fourth fault line, opposing, this time, the Sovereignty coalition to the rest of the EU. The identification of the fault lines derives from the conceptual reconstruction of the 2020 policy-making process, which started with the division on the definition of the crisis, then moved to the confrontation on the resources and their governance for going beyond the pandemic and finally ended with the clash on the rule of law’s conditions to respect for using those resources.

The 2020 EU politics was thus characterized by lasting, not shifting, coalitions. The member states of the three coalitions shared a common policy approach to the pandemic, coherently replicated in the various fault lines. With few exceptions (such as Germany, whose government moved from the Frugal to the Solidarity coalition during the crisis, or Slovenia, whose government stood initially with the Solidarity coalition, then supported the intergovernmental view of the Frugal coalition and finally backed the Sovereignty coalition on the rule of law division), the coalitions were relatively stable in their core, acted in a coordinated way, and expressed a policy view shared by their members. The analysis is based on the policy positions of national governments, not on the preferences of national public opinions. This made possible the use of the discursive institutionalism’s approach (Carstensen and Schmidt 2018; Schmidt 2015), since it explains “political and social reality (through, *ed.*) the substantive content of ideas and the interactive processes of discourse in institutional context” (Schmidt, 2015: p. 171). By political discourse, it is meant the policy rationale (which combines the general interpretation of the problem with the specific proposal for solving it) around which coalitions came to aggregate. A political discourse, however, requires political leadership for becoming a mobilizing narrative. Under the political leadership of specific national actors (the Dutch prime minister Mark Rutte, the Italian prime minister Giuseppe Conte and the French president Emmanuel Macron, and the Hungarian prime minister Viktor Orban), the three coalitions were coordinated outside and inside the European Council, thus transformed into communicative coalitions. The persistence of the three coalitions in the various fault lines is thus investigated through the method of process-tracing based on causal-process relations between temporally different policy-making phases (Beach 2017). The aim is to detect the “trajectory of change and causation” (Collier 2011: p. 823) which led to the regulation of 16 December 2020 formalizing NG-EU.

The coherent persistence of the three coalitions, along the fault lines logically structuring the 2020 policy-making process, could not be explained by the two main approaches (predominant in EU studies) to policy change. For the (liberal and new) intergovernmentalism’s approach (Moravcsik 1998; Puetter 2014), national governments might form ad hoc and minimum-sized coalitions (in the intergovernmental Council and European Council), but it is unlikely that they participate to negotiations constrained by pre-established alignments, as it happened in the various fault lines emerged during the 2020 policy-making process. Furthermore, intergovernmental negotiations generally lead to a lowest common denominator policy outcome, while NG-EU is all but a lowest common denominator policy outcome. For the (neo) functionalism’s approach (Dehousse 2011), policy change is due to the European Commission’s capacity to advance its own proposals over the resistance of member state governments. Yet, NG-EU does not emerge from an inter-institutional combat but from infra-institutional divides. Proposing NG-EU, the European Commission took sides in the already mobilized cleavage between coalitions, strengthening one coalition to the detriment of the other, rather than strengthening supranational institutions to the detriment of intergovernmental ones. Furthermore, NG-EU was decided notwithstanding the intense process of politicization surrounding the EU answer to the pandemic, contradicting the constraining dissensus’ logic as argued by post-functional scholars (Hooghe and Marks 2009). The policy outcome of the EU 2020 political process asks for a different approach for being interpreted.

The use of the coalitions’ approach, however, is here delimited to the 2020 experience. The insurgence of a new existential crisis (as it is the case with the Russian aggression of Ukraine) might trigger a decomposition of the coalitions emerged during the pandemic. One has only to think to the division between Poland and Hungary regarding the relation with Russia (thus weakening the Sovereignty coalition) or to the division between France and Germany regarding the energy’s consequences of the sanctions against Russia (thus weakening the Solidarity coalition). Moreover, a change in the government of a member state might lead to a change in the latter’s coalitional position, if not to the formation of new coalitions for dealing with new policy challenges. However, one might also argue that the three coalitions emerged during the 2020 pandemic reflected previously configured ‘sub-regions’ (Closa et al., 2016), and not occasional alliances. Several studies have indeed shown the development, within the EU, of ‘European regions’ constituted by groups of member states experiencing forms of ‘denationalization’, accompanied by process of trans-nationalization within a specific area but not supra-nationalization (Kriesi, 2016). The formation of sub-regions (Braun, 2021: 3–4) might be due to functional need (to increase the bargaining power of the concerned member states) or to identity need (to affirm the constitutional distinctiveness of the concerned member states) or both. However, it is the latter’s more than the former’s need that would transform a group of countries into a sub-region. It remains to be seen whether a similar sub-regional predisposition will persist in future critical junctures. Yet, in the 2020 policy-making process the mobilization of coherent interstate coalitions changed the dynamics of the EU policy-making process.

I will proceed as follows. I will first reconstruct the policy position of the coalitions on each of the fault lines that emerged during 2020. I will focus on national governments, using official documents, media reports and statements from various sources. Then, I will conclude discussing whether the three coalitions’ coherent persistence can be considered the development of pre-existent processes, developed during the 2010s multiple crises, leading to the construction of sub-regions as political entities, wondering whether they express a possible form of territorial segmentation of the EU political order.

## First fault line: which crisis?

The pandemic was a tremendous exogenous shock. A first division can be conceptualized concerning the interpretation of the healthcare crisis and its economic consequences. This division was brought immediately to the surface by the harsh battle between a coalition of northern member states (the Frugal coalition led by the Dutch Prime Minister Mark Rutte, with the backing of Germany) and a coalition of nine member states (the Solidarity coalition led by French President Emmanuel Macron and the Italian Prime Minister Giuseppe Conte). During the first few months of 2020, the discussion was the following: is the pandemic an asymmetric crisis affecting one or more member states (like the other crises which occurred throughout the previous decade) or is it a symmetric crisis affecting all the member states (as has never happened before)?

The battle over the interpretation of the pandemic erupted immediately, following a pattern very familiar from the multiple crises of the 2010s (particularly, at the beginning of the financial crisis in 2009–2010) (Matthijs and Blyth 2015). That is, the definition of the crisis which emerges as predominant will then affect the policy approach and instruments used to handle it (Genschel and Jachtenfuchs 2018). When the pandemic became evident in February and March 2020 in Italy and then Spain, the governments of the northern countries (the Dutch in particular), pressured by domestic Eurosceptic oppositions, immediately turned to the moral hazard paradigm to interpret its consequences. The health crisis was thus publicly presented as an asymmetric phenomenon, the dramatic effects of which (in terms of deaths and failing healthcare systems in the two southern states) were due to the lack of preparedness on their part, which found themselves facing the pandemic with high public debt (in the Italian case) or inefficient healthcare organisation (in the Spanish case). It followed (from this view) that, for the two southern countries, the handling of the crisis should be undertaken using the tools of national public policies, the effectiveness of which was increased by the temporary relaxation of the Maastricht parameters. In fact, on 23 March, the Council of Economic and Finance Ministers (the ECOFIN Council) approved the Commission’s proposal to suspend the Stability and Growth Pact (SGP) criteria which prescribe rigorous expenditure limits regarding public debt and deficit relative to national GDP.^1^ It was the first time that the SGP had been suspended, just as it was also the first time that the rules restricting state-aid to failing companies had been eased. Moreover, in those very first months of the pandemic, existing intergovernmental financial instruments were mobilised (such as the European Stability Mechanism (ESM)^2^ and the European Investment Bank (EIB)^3^), plus new and limited EU instruments were activated (such as the new programme of €100 billion for “Support to mitigate Unemployment Risks in an Emergency”, SURE). Although on 12 March the European Central Bank (ECB)’s new president, Christine Lagarde, stated that it was not the ECB’s duty to close the spread between sovereign-debt instruments of the various national governments (particularly, between German and Italian bonds), caused by the diffusion of the pandemic, on 18 March the ECB dramatically changed its policy position, announcing the creation of a new “Pandemic emergency-purchase programme” (PEPP).^4^ Thus, for moral hazard paradigm supporters, the national governments of countries worst hit by the pandemic had at their disposal national policy tools no longer constrained by SGP prescriptions, integrated by intergovernmental programmes, with the ECB powerfully engaged in covering them on the financial markets.

The moral hazard paradigm was roundly rejected by the governments of the Solidarity coalition,^5^ with the support of crucial supranational actors (the European commissioners Paolo Gentiloni and Thierry Breton and the presidents of the main parties in the EP). The dramatic experience of the sovereign debt crisis’ management during the first half of the 2010s in southern Europe led to an immediate reaction to the moral hazard paradigm. On 25 March, the leaders of nine member states of the Economic and Monetary Union (EMU) signed a letter to European Council President Charles Michel, advancing a different paradigm to interpret the health crisis. According to the signatories, the pandemic epitomised a symmetric crisis (potentially affecting all EU countries), although with asymmetric effects (some countries, those in the south, hit harder than others, those in the north), for which none of them could be considered responsible. “We are collectively accountable for an effective and united European response”,^6^ the national leaders of the nine member states wrote in the letter. According to the nine national governmental leaders, this disaster created the necessity to offer a common European response, such as issuing a common debt instrument. “This common debt instrument should have sufficient size and long maturity to be fully efficient and avoid roll-over risks now as in the future”, the nine national leaders wrote. “The funds collected will be targeted to finance in all Member States the necessary investments in the healthcare system”. Because Italy was the country first hit by the pandemic, the Italian prime minister Giuseppe Conte took the lead in the communication campaign for advancing the new interpretation. “At the end of March, he (Giuseppe Conte, *ed*) gave television and print interviews in prominent German and Dutch media outlets, making his case and allaying fears about taxpayer exposure to Italian debt” (Truchlewski et al. 2021: p. 15). In short, while these governments elaborated the discourse that a common challenge requires a common answer, for the Frugal coalition these were only national problems. The eastern countries played a secondary role in this debate, although the Solidarity coalition’s interpretation of the crisis fit better with their interests.

## Second fault line: which resources?

A second division can be conceptualized regarding the resources for building the post-pandemic recovery. Following the request advanced by the Solidarity coalition as early as 25 March to issue a common debt instrument, the French president started a ‘politics of persuasion’ (Crespy and Schramm 2021) aimed to bring the German chancellor to endorse that request in preparation of their meeting set for 18 May. Indeed, the two leaders came out from that meeting with a Recovery Fund’s proposal consisting “of €500 billion to provide EU budgetary expenditure—not loans but budgetary expenditure—for the regions and sectors most affected by the pandemic",^7^ inviting the European Commission to borrow money on financial markets to fill the €500 billion cash pot and distribute it to governments through the EU budget.^8^ The European Commission followed suit on 27 May, making the proposal its own, renaming it as ‘Next Generation EU’ and increasing it to €750 billion (two-thirds as grants, one-third as loans). It was also specified that NG-EU should support only national recovery programmes coherent with the policy priorities proposed by European Commission President Ursula von der Leyen on 16 July 2019 (when she was elected by the EP) and 27 November 2019 (when she presented the new European Commission).

In this regard, the change in position of the German government was decisive for tilting the balance on the side of the Solidarity coalition (Buti 2020). In fact, at the beginning of the pandemic, the German government reiterated its hostility towards any policy of EU indebtedness, consistent with the position held by the Frugal coalition. After just a few months, the German government dramatically reversed its position, converging towards the Solidarity coalition’s request of the previous 25 March. Several factors might be considered responsible for Merkel’s change of mind. Certainly, the power of persuasion exercised by the French president was crucial, but also internal factors (to Germany) played a role. The German chancellor soon understood that she had to pursue a ‘polity-maintenance’ approach (Ferrera et al. 2021), probably because lobbied by German industrialists worried about the disruption of southern member state economies. Also, the ruling by the German constitutional court on 5 May, which affirmed the illegitimacy (according to the German basic law or *Grundgesetz*) of the quantitative easing programme started by the ECB in 2015,^9^ led Merkel to weigh anchor from financial orthodoxy’s harbour.^10^ Finally, as Freudlsperger and Jachtenfuchs (2021: p. 118) noticed, Merkel’s decision might have reflected her tendency to support “the creation of some supranational capacity (only when that, *ed.*) appears unavoidable”. Renewed Franco-German cooperation could thus start again (Christiansen 2020), although on the bases of the Solidarity coalition’s policy proposal.

The creation of EU debt was fiercely opposed by the Frugal coalition. Immediately after the Franco-German non-paper, on 23 May,^11^ the governments of the coalition issued an alternative non-paper where it was stated that “we suggest setting up a temporary, one-off Emergency Fund to support the economic recovery and the resilience of our health sectors to possible future waves (…) What we cannot agree to, however, are any instruments or measures leading to debt mutualization nor significant increases in the EU budget”. They insisted that the EU’s help, if necessary, should be provided in the form of loans, with expiry dates within which they must be repaid and with conditions for their use.^12^ Particularly outspoken was the Dutch prime minister, Mark Rutte, also because his country was going to have parliamentary elections the following year (and the domestic Eurosceptic camp was already mobilized). However, given the scale of the pandemic, the use of loans alone would further weaken the worst affected countries, deteriorating their public finances in the medium term. Hence, the request by the Solidarity coalition to assign those resources also as grants which do not require repayment. The division over loans and grants was both economic and political. With grants, it would be possible to rebuild a level playing field among member states. With loans, it would instead be likely that the economic divergence between the least and the most affected countries would further exacerbate.

Given the spread of the virus, the Frugal coalition’s communicative discourse had to sail a stormy sea. Its leaders gradually adapted to pursue a politics of containment of the Solidarity’s coalition’s requests. They asked to reduce the amount of the NG-EU, then to increase its loan component relative to the grant component, then to financially rebalance the programme with a reduction in the MFF 2021–2027, finally proposing a slightly revised version of NG-EU on the condition of having an increase in their rebates (which were already considered unjustified by the European Commission).^13^ But they did not give up their communicative discourse, as made clear by the Dutch prime minister Mark Rutte who, in an interview to the Italian daily *Corriere della Sera* on July 2*,* argued that countries (like Italy) “next time should be able to respond to the crisis by their own means… (adding that, this time) the help should consist only of loans”.^14^ The interview was an illuminating representation of the communicative battle between the two opposed coalitions.

After the unsuccessful meeting held on 19 June 2020,^15^ it took five days (17–21 July) of bitter discussions for the European Council to agree on the Commission’s NG-EU, which followed the Franco-German proposal of setting up “an ambitious, temporary and targeted (programme, *ed*) in the framework of the next MFF”,^16^ although the agreement implied changes in the composition of the programme (which, however, remained at €750 billion) and in the size of the EU budget (to which NG-EU had to be attached). Regarding the NG-EU, its central programme, the Recovery and Resilience Facility (RRF), would provide a total of €672.5 billion to support investment and reforms. Grants worth a total of €312.5 billion would be provided to member states under the Facility, and the remaining €360 billion would be provided in loans. In the end, the €750 billion NG-EU was split into 390 billion in grants and 360 billion in loans, while it reduced the budget for a few (although significant) established programmes (in the context of the MFF 2021–2027). In the same July’s meeting, it was also agreed that the financing of NG-EU could not come from national contributions, since national budgets were already under pressure due to the pandemic. Following the request of the Solidarity coalition, NG-EU will be guaranteed through EU debt assured by the EU budget, getting the resources to pay back interest on funds for the NG-EU from new European taxes.^17^ NG-EU reflected a compromise but on the Solidarity coalition’s policy terms.

## Third fault line: which governance?

A third division can be conceptualized regarding the EU decision-making structure for governing the distribution of NG-EU funds. The Frugal coalition fought to the end to put in place a powerful supervisory mechanism, based on the European Council, to guarantee that the new resources would not be used to pay for past debts of the individual countries hit hardest by the pandemic. After all, the European Council constitutes the utmost expression of intergovernmentalism, a governance regime introduced with the 1992 Maastricht Treaty to manage policies close to national sovereignty’s sensibilities (Fabbrini 2015). Since it operates on a voluntary basis and decides unanimously, it can guarantee all national interests (Puetter 2014). By acting as the emergency power during the multiple crises in the 2010s (White 2020; Wessels 2016), the European Council has established itself as the political executive of the EU. Moreover, the other executive institution, the European Commission, was considered by the national governments of the Frugal coalition too close to the ‘federalist’ preferences of the EP for being trusted. Intergovernmental governance was also the preferred approach by eastern member states, giving their governments the possibility to use the threat of veto to prevent undesired outcomes.

The national governments of the Solidarity coalition reacted harshly to the request, by the Frugal coalition, to give full decision-making power to the European Council and the ECOFIN Council. Those leaders showed to have learned from the dramatic consequences of the intergovernmental management of the 2010s sovereign debt crisis (Schmidt, 2020). Indeed, in the southern member states, populist and anti-EU sentiments were still diffused, paralysing their governmental systems. The national leaders of the Solidarity coalition (some of them, as Angela Merkel, with a personal experience of the 2010s crisis) had thus good arguments for keeping NG-EU (and its financial arm, the RRF) within the EU budgetary process, in order to govern it through EU institutions. Connecting NG-EU to the MFF 2021–2027 would imply a prominent policy role by the European Commission, although with a limited oversight by the EP. The agreement reached, then become the basis of the regulation, recites (Conclusions A.19):

The recovery and resilience plans (submitted by the member states for receiving the funds, *ed.*) shall be assessed by the Commission within two months of the submission. (…) The assessment of the recovery and resilience plans shall be approved by the Council, by qualified majority on a Commission proposal, through an implementing act (…) The Commission shall ask the opinion of the Economic and Financial Committee (technical representatives of national treasuries, *ed.*) on the satisfactory fulfilment of the relevant milestones and targets. (…). If, exceptionally, one or more Member States consider that there are serious deviations from the satisfactory fulfilment of the relevant milestones and targets, they may request the President of the European Council to refer the matter to the next European Council. (…) This process shall, as a rule, not take longer than three months after the Commission has asked the Economic and Financial Committee for its opinion

The compromise finally reached entrusted the European Commission with distributing the resources, controlling their use by the national governments that received them and eventually deciding for their suspension. Nevertheless, the European Commission’s proposal will have to be approved by the ECOFIN Council, with the European Council intervening in case of a dispute between the two institutions. Because of the limited role assigned to the EP, this governance model might be defined as ‘constrained supranationalism’ (Buti and Fabbrini 2021).

Thus, a chain of causations may be detected between the interpretations of the crisis induced by the pandemic to the proposals for financing and governing the post-pandemic recovery. The division between coalitions crossed both the ‘control room’ (the European Council) and the ‘machine room’ (the European Commission) (Smeets and Beach 2020), being based on different policy discourses more than different institutional interests (intergovernmental or supranational). Finally, NG-EU represents a ‘leap forward’ (and not a ‘failing forward’, Jones et al. 2016; Jones 2020) in policy terms, with an unprecedented governance model for managing the post-pandemic recovery of member state economies.

## Fourth fault line: which rule of law?

Once the European Council agreed on the size and composition of both the MFF 2021–2027 and the NG-EU, it was the Council and the EP which had to transform the agreement into legal decisions. Since the consent of the EP is necessary for approval of the MFF 2021–2027, the EP immediately made clear that it would not approve it because of the cuts to crucial programmes introduced by national governments. This time an inter-institutional negotiation started between the Council (under the German biannual presidency) and the EP (under the leadership of its president, David Sassoli) which lasted ten weeks and ended with the acceptance by the Council of the EP’s request to increase the MFF 2021–2027 by €16 billion.^18^ On 5 November 2020, the Council and the EP reached provisional agreement on rule of law budget conditionality (regarding the use of both the NG-EU and the MFF 2021–2027), and then, on 10 November, they reached political agreement on the MFF 2021–2027. The political agreement, with the connected rule of law budget conditionality, was thus submitted to the Council for formal approval on 16 November. This is necessary to start the written procedure to adopt the Own Resource Decision. Since the NG-EU required an increase in the own resources of the EU to pay for it, that would have implied (Art. 311, Treaty on the Functioning of the European Union, or TFEU) unanimity by the Council and ratification by all member state parliaments. However, in the Council meeting, the representatives of Hungary and Poland withheld their consent, halting the entire budgetary process.^19^ The EP majority reacted forcefully to the veto threat by the Polish and Hungarian governments, although also within the EP (particularly within the European People’s Party) a division emerged on the rule of law issue.^20^

On the rule of law issue, the contrast between Hungary and Poland (whose prime ministers’ argument was shared also by other eastern member state prime ministers who, however, remained silent because of their concern to finally get NG-EU funds^21^) and the EU supranational institutions (European Commission and European Court of Justice or ECJ) is indeed long-lasting.^22^ The pandemic exacerbated the contrast. The governments of Hungary and Poland increased their control over the national judiciary, the media, and the political infrastructure of the opposition, in the name of the need to protect their citizens from the virus.^23^ Despite several calls from the European Commission, the two governments continued their illiberal policy in contrast with the legal principles celebrated by the Lisbon Treaty.^24^ The European Commission’s warnings were considered, by the Hungarian prime minister Viktor Orbán “an intrusion into our national sovereignty”^25^ so unacceptable to be rejected. Thus, the Polish and Hungarian prime ministers insisted to threatening the use of veto, notwithstanding it would penalise them as well (their two countries rely massively on the transfer of EU funds to support their economies).^26^ Their campaign had a powerful domestic rationale. It aimed to mobilize domestic voters around the idea of an eastern European identity “scorned” by western national leaders.

It took one month of contrasted discourses over the rule of law conditionality to reach an agreement. On the meeting of 10–11 December, the European Council concurred that, “with regard to the draft Regulation on a general regime of conditionality for the protection of the Union budget (…), the European Council underlines that the Regulation is to be applied in full respect of Article 4(2) TEU, notably the national identities of Member States inherent in their fundamental political and constitutional structures (thus stressing that, *ed.*) the guidelines will be finalised after the judgment of the Court of Justice so as to incorporate any relevant elements stemming from such judgment”. In instructing the Council and the EP, but also the ECJ, on how to interpret the Regulation on rule of law, the European Council overstretched again its role, according to a pattern already identified by Fossum (2020). The EP majority approved, on 14 December, a motion for a resolution which stated that “the European Council conclusions on the Regulation on a general regime of conditionality for the protection of the Union budget is superfluous (reminding) that the applicability, purpose, and scope of the Rule of Law Regulation is clearly defined in the legal text of the said Regulation”.^27^ Nevertheless, the European Council’s interpretation was sufficient to convince the Polish and Hungarian prime ministers to withdraw their veto threats. On 16 December, finally, with the EP's consent, the Council adopted the regulation laying down the MFF 2021–2027,^28^ together with the NG-EU recovery instrument. On these bases, the EP, the Council, and the European Commission signed an inter-institutional agreement on budgetary discipline, cooperation in budgetary matters and sound financial management, as well as on new own resources, including a roadmap towards the introduction of the latter.

Thus, although during the March–July period, the eastern member state governments took an opportunistic position in the debate, the rule of law conditionality’s issue activated the two most important eastern European governments. The Sovereignty coalition brought to the surface a division concerning the very legal nature of the latter. Whereas both the Frugal and Solidarity coalitions’ leaders consider the EU a community of law, based on the principle enshrined in the Treaties (particularly in Art. 2, Treaty on the European Union or TEU), this was not the case for the Hungarian and Polish leaders. If the previous fault lines indicated opposed policy discourses, the rule of law fault line expressed a division framed into a constitutional narrative with identity implications. To sum up, the 2020 EU policy-making process was characterized by the confrontation between three stable coalitions of countries, sharing a consistent policy rationale all along the crucial fault lines that conceptually structured the policy-making process which led to NG-EU (see Table 1).

**Table 1:** Interstate coalitions in the 2020 EU political process

Fault lines	Frugal coalition	Solidarity coalition	Sovereignty coalition
Which crisis	Asymmetric	Symmetric	Symmetric
Which resources	Ad hoc programmes	New own resources	New own resources
Which governance	Intergovernmental	Mixed (intergov./supranat.)	Intergovernmental
Which rule of law	EU treaties	EU Treaties	National constitution

## From interstate coalitions to sub-regions?

If discursive institutionalism has been useful for understanding *how* the three communicative coalitions operated, it remains to explain *why* those communicative coalitions remained so consistent along the various fault lines (as they were here conceptualized). It is arguable that the necessity to answer the pandemic’s dramatic effects activated the legacy of reciprocal cooperation between groups of member states, with the relative policy rationale. The policy rationale shared by the members of the Solidarity coalition reflected their need to stabilize the EMU, which constitutes the organizational bases for their regularized policy coordination. The policy rationale of the members of the Frugality coalition reflected their preference for intergovernmental solutions which has distant roots. Denmark (from 1960 to 1972), Sweden (from 1960 to 1995), Austria (from 1960 to 1995) and Finland (from 1961 to 1995) were members of the international organisation which was (and is) an alternative to the EU, namely the European Free Trade Association (EFTA). When they finally entered the EU, they generally positioned themselves in the Eurosceptic camp headed by the United Kingdom (an EFTA member from 1969 to 1972), declaring a primary interest to participating to the single market’s programme, although both Austria and Finland decided also to enter the EMU in 1999. Denmark, Sweden, and Finland are also members of the Nordic Council which coordinates others non-EU member states (as Iceland and Norway) participating in the European Economic Area (EEA) with the EU member states. The Netherlands, which is one of the six founding member states of the EU, has traditionally held an intergovernmental approach to an integration process interpreted mainly in economic terms. Brexit made even more urgent, for these countries, to act in a coordinated manner notwithstanding their different position regarding the EMU, with the Dutch government taking the place of the British government as leader of the group.

Also, the Sovereignty coalition was based on a long practice of reciprocal cooperation.^29^ Poland and Hungary (which, together, represent more than half the population of eastern European member states) started their cooperation in the context of the Visegrad group (constituted also by the Czech Republic and Slovakia, the V4) since 1991, first for preparing the enlargement’s negotiations and then, after 2004, for coordinating their position before the meetings of the EU intergovernmental institutions (European Council, particularly) (Kazharski 2017; Copeland 2013). However, it was the mid-2010s migration crisis that led the national leaders of the two countries to give their cooperation a clearer policy rationale. Notwithstanding important differences between the V4 (Slovakia entered the EMU on 2009, while the other states maintained their national currency), according to Bedea and Kwadwo (2021: p. 386) “the outburst of the migration crisis in 2015 (led, *ed.)* the V4 political elites to construct not only discourses but also a new regional identity in juxtaposition with promoted Western values”. The campaign against rule of law conditionality, run by the Hungarian and Polish prime ministers, was instrumental for further advancing the idea of a specific constitutional identity of eastern European countries. Using for their own ends the narrative of constitutional pluralism (expression of a liberal theory, Walker 2002, according to which each member state has its own constitutional tradition to preserve), the two leaders claimed that their own rule of law regimes, expression of their own national constitutional traditions, cannot be questioned from Brussels. Except for the few policies (i.e. mainly trade and competition) where the EU has exclusive competences, for the Polish and Hungarian governments the national constitution should be considered pre-eminent vis-à-vis EU Treaties, a claim that made possible, according to several scholars (Kelemen 2020), the authoritarian involution of those countries. The ruling of the German constitutional court on 5 May 2020 made the sovereigntist claims less eccentric than generally assumed. Although the ruling cannot be taken as a sovereigntist position, it did, however, provide the latter with hard cultural legitimacy, advancing the idea that the EU is an organization ‘under international law’.^30^

Similar interstate coalitions were formed also in previous critical phases of the European integration process, particularly during the sovereign debt crisis in the first half of the last decade (which pitted the southern against the northern member states of the EMU, with Germany leading the latter group and France trying to play the bridging role between the two coalitions) and during the migration crisis of 2015–2016 (which exacerbated the contraposition between the western and eastern European member states, with the latter coalesced against the European Commission’s proposal of automatic redistribution of refugees) (Matthijs 2020). There was certainly a degree of overlapping between the 2010s and 2020 coalitions, but also significant differences (i.e. regarding the position taken by German government). A crisis of a pandemic’s magnitude drove national leaders to rely on previous experience of ‘sub-regional cooperation’ for increasing their leverage, although different crises do not necessarily generate similar patterns. In fact, each crisis can be differently interpreted by the various members of previously formed interstate coalitions.

The article does not claim that the existence of sub-regions has become a permanent feature of the EU political process, but that interstate coalitions’ politics had, during the 2020 policy-making process, more impact on policy outcome (NG-EU) than the inter-institutional dynamics between supranational and intergovernmental actors. This signals a functioning of the EU affected not only by the interaction between intergovernmental and supranational logics, but also by the tension between sub-regions or coalitions of member states. The role played by those sub-regions in 2020 highlighted the emergence of a territorial segmentation of the EU policy-making process. In an innovative book edited by Batora and Fossum (2020: p. 7), it is argued that the EU is moving towards a segmented political order structured around three features: (1) a segmental logic triggered by a cognitive bias “that gives rise to cognitive closure and informs policy-making”; (2) “a set of organisational or procedural arrangements that sustains the segmental logic”; (3) an imbalance of the overall institutional system that allows the segments to be “systematically stronger than the de-segmenting institutional arrangements”. The outcome is the formation of segments “defined as…stable patterns of how participants, problems, solutions, and opportunities of choice are linked”. These segments, according to Batora and Fossum, cross the institutional system of the EU mainly at the horizontal level (within the Brussels’ institutions). The 2020 experience adds a further element to their picture, namely a segmentation at the vertical level as well (the one connecting member states to Brussels’ institutions). A segmentation based on distinct “cognitive biases” (or policy rationales), “organisational and procedural arrangements” (or sub-regional alliance) and a “confrontational logic” (due to opposed policy aims) challenges the consensual assumption of the intergovernmental institution (mainly the European Council) ‘fusing’ national governments and EU decision-making institutions. Will future crises confirm this territorially segmented political order?

## Conclusion

The article has argued that the 2020 EU political process brought to the surface divisions between lasting sub-regional coalitions, displaying a common policy position, at the various fault lines, framed within a shared political discourse. Through process tracing analysis of three causally correlated fault lines, the article has shown the consistency of the two coalitions of member states (the Solidarity and the Frugal coalitions) that opposed each other over the interpretation of the pandemic crisis, the resources for promoting the post-pandemic recovery and the governance of the latter. Regarding the fourth fault line (concerning the rule of law conditionality for receiving the resources of NG-EU), the article has shown how the two coalitions converged in opposing a third coalition of eastern member states (the Sovereignty coalition). Considering the inter-coalition politics emerged already during the 2010s multiple crises, the article concludes wondering whether the three coalitions signalled the existence of sub-regions, which can be activated under certain condittions, as territorial segments of the EU political order. Be as it may, an inter-coalitional (rather than intergovernmental or functional) dynamic should be taken into consideration for explaining the approval of NG-EU as the crucial programme for bringing the EU member states beyond the pandemic.

## Footnotes

1 See, https://www.consilium.europa.eu/en/press/press-releases/2020/03/23/statement-of-eu-ministers-of-finance-on-the-stability-and-growth-pact-in-light-of-the-covid-19-crisis/.

2 The harsh lending conditions imposed on Greece in the 2010s to end its debt crisis generated, however, widespread distrust towards the ESM in southern European countries (Jacoby and Hopkin 2020). Probably also for this reason, in facing the pandemic, the ESM decided to inaugurate a new line, the Pandemic Crisis Support of €240 billion, based on its Enhanced Conditions Credit Line (ECCL), available to all EMU countries, with the only condition to use the money for “direct or indirect health expenditure”. Nevertheless, no EMU member state has ever applied to benefit from the new programme of Pandemic Crisis Support.

3 The EIB mobilised €200 billion to support small and medium size enterprises.

4 The PEPP is a temporary asset purchase programme of private and public sector securities, originally consisting of €750 billion. On 4 June 2020, the ECB Governing Council decided to increase the €750 billion envelope for the PEPP by €600 billion to a total of €1,350 billion, https://www.ecb.europa.eu/mopo/implement/pepp/html/index.en.html.

5 According to *Politico* (https://www.politico.eu/article/netherlands-try-to-calm-storm-over-repugnant-finance-ministers-comments, March 29, 2020) “the Dutch financial minister Wopke Hoekstra reportedly called for Brussels to investigate why some countries did not have enough financial room for maneuver to weather the economic impact of the crisis. (This is) ‘repugnant’, a visibly irritated Costa (Prime Minister of Portugal, *ed)* declared*.* (Adding that, *ed)*’No one has any more time to hear Dutch finance ministers as we heard in 2008, 2009, 2010 and so forth’.

6 See https://balkaneu.com/eu-joint-letter-of-9-european-leaders-to-charles-michel/

7 See https://www.politico.eu/article/france-germany-propose-e500b-eu-recovery-fund/.

8 The document recites, “given the exceptional nature of the challenge of the COVID-19 pandemic for economies across the EU, France and Germany propose to allow the European Commission to finance such recovery support by borrowing on markets on behalf of the EU under the provision of a legal basis in full respect of the EU Treaty, budgetary framework and rights of national parliaments”, https://www.coronavirus-statements/article/european-union-french-german-initiative-for-the-european-recovery-from-the.

9 The German constitutional court was responding to a complaint filed in 2015 by a group of economists questioning the legitimacy of the ECB’s decision to pursue a large-scale asset purchase programme (so-called quantitative easing). The German constitutional court, first, sought guidance from the European Court of Justice (ECJ) but then refused to accept the latter’s ruling that the ECB acted within its mandate. The refusal was incendiary. The German constitutional court stated that the ECJ ruling was “incomprehensible” and in contradiction with German constitutional law, because the ECB crossed the line separating monetary from fiscal policy. Consequently, it requested the German federal government to withdraw from the quantitative easing programme, unless the ECB could explain more persuasively its rationale in the following three months, https://www.bundesverfassungsgericht.de/SharedDocs/Entscheidungen/EN/2020/05/rs20200505_2bvr085915en.html.

10 Particularly influential on the Chancellor was considered to be the interview given to *Die Zeit* by her Finance’s minister (and vice-chancellor) Olaf Scholz on 19 May, https://www.zeit.de/zustimmung?url=https%3A%2F%2Fwww.zeit.de%2F2020%2F22%2Folaf-scholz-europaeische-union-reform-vereinigte-staaten.

11 https://g8fip1kplyr33r3krz5b97d1-wpengine.netdna-ssl.com/wp-content/uploads/2020/05/Frugal-Four-Non-Paper.pdf.

12 It should be based “on a ‘loans for loans’ approach, which is in line with fundamental principles for the EU budget”, https://g8fip1kplyr33r3krz5b97d1-wpengine.netdna-ssl.com/wp-content/uploads/2020/05/Frugal-Four-Non-Paper.pdf.

13 In the MFF 2014-2020, Denmark, the Netherlands and Sweden already benefitted from gross reductions in their annual GNI contribution, respectively, of EUR 130 million, EUR 695 million and EUR 185 million (expressed in 2011 prices). Austria benefited from a gross reduction in its annual GNI contribution until 2016. This practice was harshly criticised by the European Commission, see https://ec.europa.eu/info/strategy/eu-budget/revenue/own-resources/correction-mechanisms_en.

14 https://www.corriere.it/sette/esteri/20_luglio_02/rutte-l-italia-deve-imparare-farcela-sola-7b5ec844-bbda-11ea-8b38-f3e0b4a46805.shtml.

15 European Council President Charles Michel remarked, at the end of the meeting, that, “on different topics we observe that it is necessary to continue to discuss”, 19 June 2020, https://www.consilium.europa.eu/en/press/press-releases/2020/06/19/remarks-by-president-charles-michel-after-video-conference-of-the-members-of-the-european-council-19-june-2020/.

16 See https://www.coronavirus-statements/article/european-union-french-german-initiative-for-the-european-recovery-from-the.

17 Four new taxes were indicated by the European Commission: emissions trading system-based own resources; own resource based on operations of companies that draw huge benefits from the EU single market; carbon border adjustment mechanism; digital tax on companies with a global annual turnover of above €750 million. According to the European Commission, “taken together, these new own resources could help finance the repayment of and the interest on the market finance raised under Next Generation EU”, https://ec.europa.eu/info/sites/info/files/factsheet_3_04.06.pdf.

18 See, https://www.europarl.europa.eu/news/en/press-room/20201106IPR91014/compromise-on-long-term-eu-budget-ep-obtains-EU16-billion-more-for-key-programmes/.

19 The MFF 2021–2027 requires the unanimous vote of the Council, after receiving the EP’s consent, Art. 312 TFEU, while the NG-EU is co-decided by the Council and EP, but it requires the unanimous approval of the Own Resource Decision by the Council and all member state parliaments, Art. 311 TFEU.

20 Indeed, a MEP from the Fidesz party, Tamas Deutsch, compared the leader of his own EP parliamentary party (EPP), Manfred Weber, “to a Gestapo officer” for his insistence on defending rule-of-law conditionality. Tamas Deutsch was then suspended by the party, although a final decision on his membership was postponed.

21 According to the European Commission’s 2020 Rule of Law Report, also Bulgaria, Croatia, Romania, Slovakia (and Malta as well) presented important deficiencies in their system of rule-of-law. See, https://ec.europa.eu/info/publications/2020-rule-law-report-communication-and-country-chapters_en.

22 The European Commission issued four recommendations regarding infringements of the rule of law mechanism by the Polish government between 2016 and 2018, activated infringement procedures against Hungary (Case C-286/12) and Poland (Case C-192/18, Case C-619/18 and the case filed on 29 October2020), and many other cases were brought before the ECJ regarding both countries, see the complete report by the EP, https://www.europarl.europa.eu/RegData/etudes/BRIE/2019/642280/EPRS_BRI(2019)642280_EN.pdf.

23 Daniel Kelemen, ‘Hungary just became a coronavirus autocracy’, *Washington Post,* 2 April 2020, https://www.washingtonpost.com/politics/2020/04/02/hungary-just-became-coronavirus-autocracy/.

24 See Federico Fabbrini and Daniel R. Kelemen, ‘With one court decision, Germany may be plunging Europe into a constitutional crisis’, *Washington Post,* 7 May 2020, https://www.washingtonpost.com/politics/2020/05/07/germany-may-be-plunging-europe-into-constitutional-crisis/.

25 See https://www.euractiv.com/section/economy-jobs/news/eu-leaders-to-decide-on-compromise-to-unblock-eu-budget-package/.

26 The two leaders displayed a significant difference in the tone of their threat. While the Hungarian Viktor Orbán insisted to affirm that his country could do also without NG-EU funds, the Polish Mateusz Morawiecki was less adversarial towards the European Commission, since his country was expected to be among the top beneficiaries of NG-EU. See, ‘Prime Minister Viktor Orbán’s address to the Hungarian parliament before the start of daily business’, 21 September 2020, http://abouthungary.hu/speeches-and-remarks/prime-minister-viktor-orbáns-address-to-the-hungarian-parliament-before-the-start-of-daily-business/.

27 See https://www.europarl.europa.eu/doceo/document/B-9-2020-0428_EN.html.

28 See https://www.consilium.europa.eu/en/press/press-releases/2020/12/17/multiannual-financial-framework-for-2021-2027-adopted/.

29 Although the concept of ‘sovereignism’ continues to be widely debated in the literature (see, recently, Fabbrini 2019), here it is used for indicating the view that the integration process should stop at the door of national sovereignty (whatever that means).

30 In answering the European Commission’s criticism of the Polish government, regarding the setting up of a disciplinary chamber aiming to control Poland’s Supreme Court judges, the Polish European Affairs minister Konrad Szymanski listed “judgements from other European Court, including Germany’s Federal Constitutional Court, to argue that EU law does not always have primacy” (*Politico Playbook,* 18 August 2021).
